# The Prevalence and Risk Factors of Pregnancy-Related Anxiety in Bahrain

**DOI:** 10.7759/cureus.57404

**Published:** 2024-04-01

**Authors:** Abrar M Alnasheet, Nada A Abdulaal, Nahid Kamal

**Affiliations:** 1 Obstetrics and Gynecology, Salmaniya Medical Complex, Manama, BHR; 2 Obstetrics and Gynecology, Al Kharj Military Industries Corporation Hospital (AKMICH), Riyadh, SAU

**Keywords:** maternal stress, bahrain, antenatal care visits, maternal mental health, pregnancy-related anxiety

## Abstract

Objectives

We aim to estimate the prevalence of anxiety among pregnant women, explore the possible risk factors, and compare the presence of anxiety in each gestational trimester in all pregnant women attending the antenatal care clinics at a tertiary care hospital in Manama, Bahrain.

Methods

This study followed a cross-sectional research design at the antenatal clinics of Salmaniya Medical Complex in Manama, Bahrain. Direct interviews with 513 participants were conducted using the Pregnancy Anxiety Questionnaire-Revised-2 (PRAQ-R2).

Results

Most participants (63%) were 25-35 years old. The majority (85.6%) were Bahraini nationals, and 52.2% were university-educated. Almost two-thirds were unemployed, 28.1% had associated chronic comorbidities, 3.1% had associated psychiatric disorders, 15% had a high level of anxiety, and 38% had a moderate level of anxiety. Employed participants had a significantly higher level of anxiety (p=0.022) than housewives/unemployed participants. Participants' levels of anxiety differed significantly according to their gestational age (p=0.043), with the highest anxiety among those in their third trimester (15.7%). Participants' anxiety levels were significantly higher among those with previously complicated pregnancies (p=0.002). Moreover, those with unplanned current pregnancy had significantly higher anxiety levels (p=0.019).

Conclusions

This study showed that anxiety seems to be a common disorder among pregnant women in Bahrain. It was more prevalent during the third trimester, and its occurrence was associated with the pregnant woman's employment, the occurrence of previously complicated pregnancies, and unplanned current pregnancies.

## Introduction

Anxiety is the most common psychological health problem faced by women during their pregnancies. In the Diagnostic and Statistical Manual of Mental Disorders, Fifth Edition (DSM-V), generalized anxiety is defined as excessive worry, disproportionate to current events, which the person finds difficult to control, causing distress and resulting in decreases in occupational and social functioning. During pregnancy, there are several physiological, hormonal, psychological, and social changes, associated with increased risk of emotional suffering and psychiatric morbidity [1]. It is a time of considerable transformation for women.

Although pregnancy is usually received with action and self-fulfillment, some women may experience variable levels of anxiety [2].

Harpel [3] described pregnancy-related anxiety as a common experience for a woman anticipating childbirth. Low levels of anxiety can be beneficial to prepare her for parenthood. However, anxiety may become uncontrollable and lead to adverse behavioral outcomes, necessitating thorough evaluation to ascertain the presence of potential psychiatric disorders [4].

Huizink et al. [5] noted that about 15% of pregnant women experience high levels of anxiety during this important transitional phase. They may experience worries regarding their upcoming labor, anticipated pain, fear of childbirth, concerns about the health of their baby, or the physical changes they are experiencing.

Anxiety is the most common psychological health problem faced by women during their pregnancies [6]. The current diagnostic criteria distinguish "normal" levels of worry from a generalized anxiety disorder, which is described as excessive non-specific worry, disproportionate to current events, that the person finds difficult to control, leading to distress, which decreases occupational and social functioning [7].

For some women, pregnancy-related risks and complications can be highly anxiety-provoking [8]. High-risk pregnancies [9], miscarriage [10], and previous pregnancy or birth-related complications have been frequently reported to increase the likelihood of experiencing anxiety during pregnancy [11].

High levels of anxiety may be associated with adverse health effects on the mother and the baby. Several studies reported that anxiety during pregnancy is a strong predictor for increased risk of postnatal mental health problems, disturbed infant-mother attachment relationships, and increased production of cortisol during pregnancy, which negatively affects fetal neurodevelopment and the infant's psychological development after birth [12,13].

Moreover, high levels of pregnancy-related anxiety have been related to preterm labor and premature babies [14] and a range of adverse childhood outcomes, including negative emotionality [15], attention deficit hyperactivity disorder [12], and developmental delays, as well as changes in brain grey matter volume [16]. These adverse outcomes denote that prenatal assessment of anxiety is essential to identify those with significantly increased levels of anxiety during their pregnancy and facilitate prevention and intervention efforts to reduce anxiety during pregnancy, with potentially long-term beneficial effects on the child [17].

Despite the harmful impact of anxiety on pregnancy, the mental health of pregnant women during pregnancy has received less attention than that after delivery [18]. Investigations on the incidence of anxiety during pregnancy and its associated factors remain scarce, the current understanding of antenatal anxiety remains scanty, and the current understanding of antenatal anxiety remains limited [19].

Moreover, although much progress has been achieved in studying anxiety during pregnancy, there are difficulties in identifying anxiety during pregnancy [20]. Anxiety symptoms may be frequently overlooked or even misinterpreted as "normal" pregnancy symptoms. Weisberg and Paquette [21] explained that some physiological symptoms of pregnancy may look like anxiety symptoms. For example, physical symptoms, such as tiredness, pain, or vertigo, are common during pregnancy, as well as during mental stress, which complicates screening for anxiety. Therefore, the risk of false indications of anxiety during pregnancy may be present [22].

More research is needed to understand how antenatal anxiety is experienced to improve the identification of women who might benefit from antenatal psychological support. Moreover, exploring the factors associated with antenatal anxiety may help in developing screening strategies to identify the risk groups of women who need intervention during their pregnancies.

Therefore, the present study aimed to assess the prevalence of pregnancy-related anxiety, explore related risk factors, and compare anxiety levels during each gestational trimester in women attending the antenatal care clinics at a tertiary care hospital in Manama, Bahrain.

## Materials and methods

Following a cross-sectional research design, the present study was conducted at the antenatal clinics of Salmaniya Medical Complex. It is the largest hospital in Bahrain and is located in the capital city, Manama. The hospital provides a wide range of medical services and specialties, including emergency care, surgery, obstetrics and gynecology, and pediatrics. It is a government-funded hospital and is a major healthcare facility in Bahrain. It plays a crucial role in providing healthcare services to the residents of Bahrain and also serves as a teaching hospital affiliated with the Arabian Gulf University.

The study was conducted from June to August 2023. The inclusion criteria were all pregnant women attending the antenatal clinics at the study hospital who agreed to participate in the study. The collected data included participants' personal characteristics, obstetric history, and past history of anxiety. The researchers also conducted direct interviews with participants using the Pregnancy Anxiety Questionnaire-Revised-2 (PRAQ-R2). Following a consecutive sampling, a total of 513 pregnant women were included in this study. The PRAQ-R2 is a 10-item self-report questionnaire that measures pregnancy-specific anxiety. It is appropriate for both primiparous and parous women. Items are answered on a 5-point Likert scale ranging from 1 to 5, with higher scores reflecting greater anxiety levels [5].

Ethical approval for conducting the present study was obtained from the Research Ethics Committee of Government Hospitals (IRB approval number: 52230523, date: 23/5/2023). Informed consent to participate in the study was obtained prior to personal interviews. Confidentiality and privacy were ensured during all steps of this study.

## Results

Table 1 shows that the age of most participants (63%) were 25-35 years old. The majority of participants (85.6%) were Bahraini nationals. More than half of the participants (56.9%) lived within nuclear families. More than half of the participants (52.2%) were university-educated, while 41.3% had school-level education. Almost two-thirds were unemployed. About one-quarter (28.1%) had associated chronic comorbidities (e.g., diabetes and hypertension), while 3.1% had associated psychiatric disorders.

**Table 1:** Personal characteristics of the study participants

Personal characteristics	Number	%
Age groups		
<25 years	71	13.8
25-35 years	323	63.0
>35 years	119	23.2
Nationality		
Bahraini	439	85.6
Non-Bahraini	74	14.4
Type of family		
Nuclear	292	56.9
Extended	221	43.1
Educational level		
Illiterate	21	4.1
School	212	41.3
University	268	52.2
Postgraduate	12	2.3
Employment status		
Housewife/unemployed	328	63.9
Employed	185	36.1
Associated chronic comorbidity	144	28.1
Associated psychiatric disorders	16	3.1

Table 2 shows that 23.4% of participants were primigravida, while 56.1% were gravida (2-4). Most participants had <4 previous deliveries, while 11.5% had four or more previous deliveries. About two-thirds of participants had no previous abortions, while 22.8% had a past history of one abortion, 7.4% had two abortions, and 3.9% had three or more previous abortions. About two-thirds of participants (64.7%) were in their third trimester, while 21.8% were in their second trimester, and 13.5% were in their first trimester. Almost one-third of participants (31.2%) had a past history of complicated pregnancies, and 37.2% had a complicated current pregnancy. The current pregnancy of more than half of the participants (59.3%) was planned.

**Table 2:** Obstetric history of the study participants

Obstetric history	Number	%
Gravidity		
1	120	23.4
2-4	288	56.1
>4	105	20.5
Parity		
<4	454	88.5
4+	59	11.5
Number of previous abortions		
0	338	65.9
1	117	22.8
2	38	7.4
3+	20	3.9
Gestational trimester		
First trimester	69	13.5
Second trimester	112	21.8
Third trimester	332	64.7
History of previously complicated pregnancies	160	31.2
Complicated current pregnancy	191	37.2
Planned current pregnancy	304	59.3

**Figure 1:**
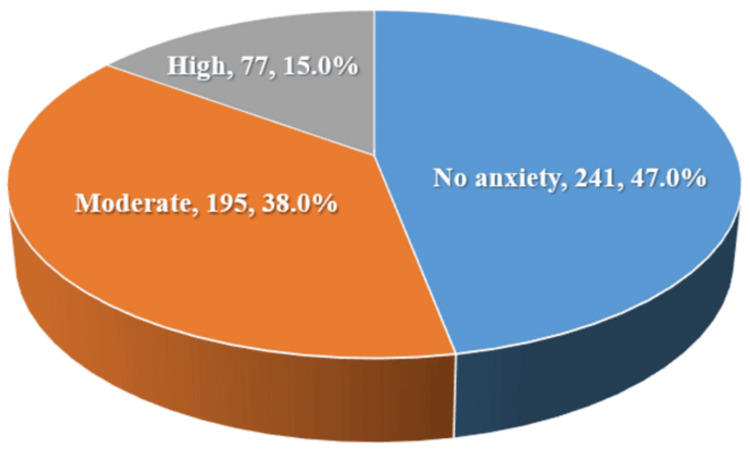
Participants' anxiety levels

Table 3 and Figure 1 show that 15% of participants had high levels of anxiety, while 38% had moderate levels of anxiety.

**Table 3:** Participants' anxiety levels

Anxiety levels	Number	%
No anxiety	241	47
Moderate	195	38
High	77	15

Table 4 shows that employed participants had a significantly higher level of anxiety (p=0.022) than housewives/unemployed participants. However, their anxiety levels did not differ significantly according to their nationality, type of family, educational level, associated chronic comorbidity, or psychiatric disorders.

**Table 4:** Participants' anxiety levels according to their personal characteristics †Statistically significant

Personal characteristics	No anxiety		Moderate		High		p-value
	Number	%	Number	%	Number	%	
Age groups							
<25 years	40	56.3	23	32.4	8	11.3	
25-35 years	146	45.2	124	38.4	53	16.4	0.462
>35 years	55	46.2	48	40.3	16	13.4	
Nationality							
Bahraini	205	46.7	168	38.3	66	15.0	
Non-Bahraini	36	48.6	27	36.5	11	14.9	0.949
Type of family							
Nuclear	136	46.6	115	39.4	41	14.0	
Extended	105	47.5	80	36.2	36	16.3	0.676
Qualification							
Illiterate	12	57.1	7	33.3	2	9.5	
School	110	51.9	72	34.0	30	14.2	
University	115	42.9	111	41.4	42	15.7	0.394
Postgraduate	4	33.3	5	41.7	3	25.0	
Employment status							
Housewife/unemployed	169	51.5	115	35.1	44	13.4	
Employed	72	38.9	80	43.2	33	17.8	0.022†
Associated chronic comorbidity							
No	182	49.3	135	36.6	52	14.1	
Yes	59	41.0	60	41.7	25	17.4	0.226
Associated psychiatric disorders							
No	237	47.7	187	37.6	73	14.7	
Yes	4	25.0	8	50.0	4	25.0	0.181

Table 5 shows that participants' levels of anxiety differed significantly according to their gestational age (p=0.043), with the highest anxiety among those in their third trimester (15.7%). Participants' anxiety levels were significantly higher among those with previously complicated pregnancies (p=0.002). Moreover, those with unplanned current pregnancy had significantly higher anxiety levels (p=0.019). However, their anxiety levels did not differ significantly according to their gravidity, parity, previous abortions, or complicated current pregnancy.

**Table 5:** Participants' anxiety levels according to their personal characteristics †Statistically significant

Obstetric history	No anxiety		Moderate		High		p-value
	Number	%	Number	%	Number	%	
Gravidity							
1	54	49.5	40	36.7	15	13.8	
2-4	135	45.3	117	39.3	46	15.4	0.927
>4	52	49.1	38	35.8	16	15.1	
Parity							
<4	215	47.4	172	37.9	67	14.8	
4+	26	44.1	23	39.0	10	16.9	0.859
Number of previous abortions							
0	150	44.4	138	40.8	50	14.8	
1	59	50.4	39	33.3	19	16.2	
2	21	55.3	14	36.8	3	7.9	0.281
3+	11	55.0	4	20.0	5	25.0	
Gestational trimester							
First trimester	28	40.6	32	46.4	9	13.0	
Second trimester	43	38.4	53	47.3	16	14.3	0.043†
Third trimester	170	51.2	110	33.1	52	15.7	
Previously complicated pregnancies							
No	183	51.8	126	35.7	44	12.5	
Yes	58	36.3	69	43.1	33	20.6	0.002†
Complicated current pregnancy							
No	163	50.6	118	36.6	41	12.7	
Yes	78	40.8	77	40.3	36	18.8	0.054
Planned current pregnancy							
No	83	39.7	88	42.1	38	18.2	
Yes	158	52.0	107	35.2	39	12.8	0.019†

## Discussion

The present study showed that 15% of pregnant women attending the antenatal care clinics had a high level of anxiety. The prevalence of high levels of anxiety increases significantly among pregnant women according to their gestational trimesters (p=0.043), being 13% during the first trimester and 14.3% during the second, reaching its highest prevalence during the third trimester (15.7%). The anxiety grades of participant pregnant women did not differ significantly according to their gravidity (p=0.927) or parity (p=0.859).

The findings of this study denote that anxiety is common among pregnant women in Bahrain. Therefore, the consequences of experiencing anxiety during pregnancy on both the mothers and their babies necessitate exploring the potential complications associated with anxiety during pregnancy.

Different studies reported several risk factors for pregnancy-related anxiety. The systematic review by Biaggi et al. [18] concluded that risk factors for pregnancy-related anxiety include the lack of social support, a history of mental illness, unplanned pregnancy, adverse life events, and complications of pregnancy complication.

Rubertsson et al. [22] noted that women who suffer from anxiety before pregnancy had an increased risk of experiencing pregnancy as an especially negative and stressful event. A history of anxiety is a significant factor in anxiety among pregnant women. Moreover, the age of the mothers, particularly younger mothers, was also associated with high anxiety levels during pregnancy. The study explained the lack of association between anxiety and previous obstetric history by that the development of anxiety during pregnancy can negatively affect women regardless of the number of their children.

Our results are in agreement with those reported by Dennis et al. [23], who stated that during their pregnancies, almost 15% of women undergo generalized anxiety. The systematic review by Murray [24] reported that the overall prevalence of pregnancy-related anxiety was 14.1%. In Norway, Berle et al. [25] reported that pregnancy-related anxiety in all three trimesters of pregnancy was 10.4%. Teixeira et al. [26], in Oporto, Portugal, reported that the prevalence of pregnancy-related anxiety was 18.2% during the third trimester, being higher than those during the first or second trimesters (15% and 12.3%, respectively). Similarly, in Kerala, India, Madhavanprabhakaran et al. [27] noted that the highest prevalence of pregnancy-related anxiety was during the third trimester. In Brazil, Silva et al. [28] reported that 26.8% of pregnant women had anxiety, with the highest prevalence during the third trimester (42.9%).

Based on these results, it may be suggested that, with the advancing pregnancy, women progressively develop more severe symptoms. During their last trimester, they become more worried and anxious than during their early pregnancy. This may be further explained by that pregnant women's fears about the approaching birth may provoke the development of anxiety during late pregnancy. Therefore, risk factors for pregnancy-related anxiety should be regularly assessed by obstetricians, and where appropriate, referral to a psychiatrist should be considered. However, in some developing countries, where this may not be feasible, the role of clinical psychologists and midwives has been suggested to develop their skills to conduct screening for anxiety [27]. Moreover, physicians at antenatal clinics should provide formal routine childbirth health education to all pregnant women to lower pregnancy-related anxiety. Furthermore, since antenatal anxiety constitutes a risk factor for both postnatal anxiety, monitoring and, if required, treatment of anxiety are essential to resolve its impact on both the mother and her child.

Our study revealed that high levels of anxiety during pregnancy were significantly associated with women's employment (p=0.022), previously complicated pregnancies (p=0.002), and unplanned current pregnancies (p=0.019). Therefore, management approaches may include a combination of pharmacotherapy, psychotherapy, social support interventions, lifestyle modifications, and stress management techniques tailored to address the unique biological, psychological, and social factors contributing to each individual's experience of pregnancy-related anxiety. Moreover, healthcare providers can promote preventive measures such as early identification of risk factors, routine screening for anxiety symptoms during prenatal visits, and promoting healthy coping strategies to mitigate the negative impact of antenatal anxiety on maternal and fetal well-being.

Madhavanprabhakaran et al. [27] urged that obstetricians should give special attention to decreasing pregnancy-related anxiety. Also, they discussed that, compared to the extended family system, the nuclear family nature prompted reduced exposure to traditional knowledge transfer from the mothers to their daughters. This lack of information on childbirth preparation contributes to increased pregnancy-related anxiety. Moreover, women may look for information through newspapers or friends, which is not comprehensive or complete. Hence, it usually triggers their pregnancy-related anxiety.

Silva et al. [28], in Brazil, reported that pregnancy-related anxiety is significantly higher during the third trimester and among employed mothers than among housewives (p=0.040), those who had complications during their previous pregnancies (p<0.001), those with past history of abortion (p=0.020), and those with maternal desire regarding the pregnancy (p=0.010).

In Brazil, Silva et al. [28] noted that the incidence of anxiety during pregnancy significantly increases in women with social disadvantage, past history of abortion or fetal loss, preterm delivery, exposure to stressful events, a previous history of psychiatric illness, or treatment during previous pregnancies.

Tarafa et al. [29], in southwest Ethiopia, reported a high prevalence of pregnancy-related anxiety (32.7%). It was significantly associated with mothers' young age (<24 years old), low family income, poor social support, and unwanted pregnancy.

Also, Molarius et al. [30] noted that the highest frequency of self-reported anxiety was found in women in the childbearing age group of 18-29 years. Andersson et al. [31] added that almost twice as many pregnant women with any psychiatric complaints report noticeable fears of childbirth, compared with those who did not have any psychiatric disorder.

Evidence has been established regarding the association between high levels of anxiety during pregnancy and adverse labor outcomes [32-34]. Hence, antenatal care must focus on measures to reduce pregnancy-related anxiety.

However, the present study showed that participants' anxiety levels did not differ significantly according to their nationality (i.e., Bahraini or non-Bahraini), family type (i.e., extended or nuclear), educational level, associated chronic comorbidity (e.g., diabetes or hypertension), or associated mental disorders, in addition to some obstetric characteristics (i.e., gravidity, parity, previous abortions, or complicated current pregnancies).

This study assessed anxiety among pregnant women, which should be detected and managed early. However, this study was conducted in one center, i.e., Salmaniya Medical Complex, in Manama, Bahrain. Moreover, the study followed a cross-sectional research design, which is good for hypothesis generation rather than hypothesis testing. Therefore, it may not be easy to generalize the results of this study to other populations.

The non-significant association of each of these variables with pregnancy-related anxiety may be explained differently. The lack of difference between Bahraini and non-Bahraini participants reflects the universal accessibility of healthcare services in Bahrain. Regarding family type, the lack of significant association with pregnancy-related anxiety reflects the insignificant social support provided by extended families to pregnant women. The lack of a significant association between participants' education level or associated comorbidity with anxiety levels reflects the common need among participants to receive health education regarding the stresses of pregnancy regardless of their qualification or physical health status.

To the best of our knowledge, this study is the first to explore the prevalence of anxiety among pregnant women in Bahrain. The findings of the present study indicate the need to implement preventive interventions to decrease the incidence of pregnancy-related anxiety and, hence, positively improve birth outcomes. However, there are a few limitations to this study. It was conducted in one center, i.e., Salmaniya Medical Complex, in Manama, Bahrain. Also, the study followed a cross-sectional research design, which is good for hypothesis generation rather than hypothesis testing. Therefore, it may not be easy to generalize the results of this study to other populations.

## Conclusions

In summary, this study aimed to estimate the prevalence of anxiety among pregnant women in Bahrain and explore its associated risk factors. With the progress of pregnancy, the chances of developing anxiety become higher. About half of pregnant women had varying levels of anxiety. High levels of pregnancy-related anxiety were observed among the employed, those in their third trimester, those with previously complicated pregnancies, and those with unplanned current pregnancies. Therefore, childbirth health education should be provided to ensure well-informed and empowered expectant mothers and reduce pregnancy-related anxiety. During routine antenatal care visits, healthcare providers should promote preventive measures by early identification of risk factors, routine screening for anxiety symptoms during prenatal visits, and encouraging coping strategies to reduce the impact of antenatal anxiety.
